# Exploring longitudinal trends and multifactorial correlations of COVID-19 vaccination willingness among healthcare workers in China: a two-phase cross-sectional study before and after the 2023 phase of COVID-19 pandemic

**DOI:** 10.3389/fpubh.2025.1699531

**Published:** 2025-11-10

**Authors:** Xinyu Liu, Lin Li, Rongrong Dai, Shuhan Zheng, Ying Su, Lingling Ding, Jianyun Long, Hangjie Zhang

**Affiliations:** 1 School of Public Health, Hangzhou Medical College, Hangzhou, China; 2 Yuhang Center for Disease Control and Prevention (Health Supervision Institute), Hangzhou, China; 3 Center for General Practice Medicine, Department of Research Administration Dept, Zhejiang Provincial People's Hospital (Affiliated People's Hospital), Hangzhou Medical College, Hangzhou, China; 4 Department of Immunization Program, Zhejiang Provincial Center for Disease Control and Prevention, Hangzhou, China; 5 Department of Vascular Surgery, Hangzhou First People's Hospital, Hangzhou, Zhejiang, China; 6 Department of Prevention and Control of Infectious Disease, Key Lab of Vaccine, Zhejiang Provincial Center for Disease Control and Prevention, Hangzhou, China

**Keywords:** COVID-19 vaccine, willingness, healthcare workers, pandemic, booster dose, routine vaccination

## Abstract

**Background:**

Healthcare workers' (HCWs') acceptance of the coronavirus disease 2019 (COVID-19) vaccines constitutes a globally concerned issue, which exhibits variations across different countries and distinct phases of the pandemic. Specifically, the characteristics of HCWs' vaccine acceptance in China following the pre- and post-pandemic period in 2023 remain unclear.

**Methods:**

We conducted a population-based two-phase cross-sectional study in Zhejiang, China. Phase 1 was implemented during the pre-pandemic period (July–September 2022), with the primary objective of investigating HCWs' willingness to receive the fourth dose of COVID-19 vaccine. Phase 2, conducted in the post-pandemic period (November 2023–January 2024), focused on exploring HCWs' willingness to undergo annual COVID-19 vaccination. A total of 1,657 HCWs participated in the survey, where data on demographic characteristics, vaccination history, risk perception, and behavioral factors were collected. Multivariate logistic regression analysis was subsequently performed to identify factors associated with HCWs' vaccination willingness.

**Results:**

In the first phase (2022, *n* = 820), 70.61% of participants expressed willingness to receive the fourth dose of the COVID-19 vaccine. Separately, in the second phase (2023, *n* = 837), the willingness of HCWs to undergo annual COVID-19 vaccination was 28.43%. Concurrently, a substantial decline in HCWs' trust in vaccines was observed: 85.24% of respondents endorsed vaccine safety in 2022, whereas this proportion fell to 57.23% in 2023. Similar downward trends were noted in perceived efficacy (from 78.78% in 2022 to 53.41% in 2023) and vaccine recommendation rates (from 86.10% in 2022 to 56.27% in 2023). Women were less likely to accept a fourth dose [odds ratio (OR) = 0.56, 95% confidence interval (CI): 0.37–0.85]. Laboratory personnel (OR = 3.57, 95% CI: 1.22–10.45) and those confident in vaccine efficacy (OR = 5.06, 95% CI: 1.95–13.08) were more likely to receive a booster. Prior vaccination plus high trust strongly predicted willingness for annual vaccination adherence (OR = 7.23, 95% CI: 4.05–12.92). Despite high primary-series (99.3%) and booster (92.8%) coverage, 7.2% remained unboosted; 33.3% cited lack of awareness and 50% reported contraindications.

**Conclusion:**

Following the COVID-19 pandemic, notable shifts were observed in HCWs regarding their perceptions, acceptance, and recommendation behaviors toward COVID-19 vaccines. Collectively, the observations presented herein provide empirical evidence to inform the optimization of COVID-19 vaccination strategies targeting HCWs.

## 1 Introduction

The coronavirus disease 2019 (COVID-19) pandemic has progressed through multiple phases, and the World Health Organization (WHO) no longer classifies it as a global emergency. According to global vaccination statistics, 67% of the world population had completed primary COVID-19 vaccination series by December 2023 (1). Mass vaccination has reduced severe outcomes worldwide, while widespread vaccination remains an ongoing process in many low and middle income nations (2).

Healthcare workers (HCWs) play a dual role as frontline responders and vaccination advocates. Their immunization behaviors influence both personal and public health outcomes (3). In Saudi Arabia, a study found that the most common sources of vaccine-related information were public campaigns (36%) and HCWs themselves (36%) (4). Similarly, research in the United States has confirmed that recommendations from HCWs strongly influence public vaccination decisions (5). Furthermore, a study in Italy highlighted the effectiveness of HCWs in countering vaccine misinformation (6). As both key vaccine recipients and influential advisors, their vaccination attitudes are also reflected in their family roles. This dual capacity underscores the broad implications of their vaccine confidence.

A global meta-analysis found that HCWs had the lowest COVID-19 vaccine acceptance rates worldwide (59.77%) (7).

A multinational study including 23 countries revealed that approximately 15% of HCWs were hesitant about COVID-19 vaccination (8). An international narrative review further indicated that between 2.8% and 26% of HCWs were hesitant about booster doses (9). Beyond these heterogeneous acceptance rates, the literature consistently points to common underlying drivers of global vaccine hesitancy among HCWs. The most prominent concerns are apprehensions about vaccine safety and adverse reactions, as well as a lack of confidence in healthcare policies and institutions (9). In high income countries (e.g., Norway, the US, the UK, and Australia), vaccine hesitancy often stems from misinformation, conspiracy theories, and distrust in healthcare policies (10). In contrast, in some European regions (e.g., Croatia, France, Greece, and Romania), such hesitancy stems more from distrust in health authorities and pharmaceutical companies (11). In low and middle income countries (such as those in Asia, Africa, and South America), hesitancy is frequently associated with doubts about the necessity and effectiveness of vaccines within their local epidemiological and educational context, accompanied by heightened concerns regarding safety and side effects (12). Given that vaccine hesitancy varies across nations due to specific contexts, a pivotal question arises: within the evolving context of China's COVID-19 control measures, what factors influenced vaccine willingness among its HCWs?

The vaccination campaign for HCWs in China was both distinct and rapidly evolving, combining high primary-series coverage, evolving policy guidance, and the professional group's pivotal role (13). The first phase of our study was conducted during the implementation of the 'Dynamic Zero-COVID' policy (14). Under this policy, China has classified COVID-19 as a Category B infectious disease but managed under Category A protocols, local authorities were empowered to impose lockdowns and other restrictive measures. During this same period, high coverage of the primary COVID-19 vaccination series had been achieved, with inactivated vaccines (e.g., BBIBP-CorV and CoronaVac) as the predominant formulations, and booster vaccination campaigns were underway (15). The national vaccination program was based on voluntary participation. HCWs were identified as a priority group for early vaccination (16). The second phase of our investigation was conducted after December 2022, when China optimized its COVID-19 response by transitioning the management to standard Category B protocols. This shift brought the disease into alignment with others in the same category, such as HIV and avian influenza, marking the country's entry into a phase of routine epidemic control (17). This period was followed by official announcements on future booster strategies, which were refined to focus on voluntary annual vaccination primarily targeting older and vulnerable groups (18). The dynamic environment of policy adjustment and changing population immunity in China allowed for the exploration of the characteristics and influencing factors of HCWs' COVID-19 vaccination willingness across two distinct phases.

Separate literature from various stages of the vaccine rollout documents the levels of vaccination willingness among Chinese HCWs. Prior to the general release of COVID-19 vaccines, two studies separately reported acceptance rates among Chinese HCWs of 73.1% (19) and 79.1% (20). Following the launch of the national vaccination program in March 2021, subsequent studies documented willingness rates of 80.8% (21) and 93.9% (22), respectively. Later, a survey in August 2022 found that 76.4% of HCWs supported heterologous boosters (23). More recently, a 2023 survey indicated that 57.8% of HCWs were willing to receive a second booster dose (24). Following these earlier findings, our study aimed to explore the characteristics of and factors influencing HCWs' vaccination willingness during two subsequent critical phases: the late pandemic (2022) and the post-pandemic period (2023–2024).

We conducted a two-phase study: Phase 1 (July–September 2022) assessed fourth-dose booster willingness among HCWs in Zhejiang Province and identified factors associated with it using multivariate regression analysis; Phase 2 (November 2023-January 2024) identified factors associated with willingness to receive annual vaccination. These findings will provide a critical basis for tailoring future immunization programs for HCWs.

## 2 Materials and methods

### 2.1 Study design and participants

This study utilized stratified sampling based on geographical location, selecting one prefecture-level city each from eastern, western, southern, northern, and central Zhejiang: Jiangshan City (western Zhejiang), Shengsi County (eastern Zhejiang), Shengzhou City (central Zhejiang), Yuhuan City (southern Zhejiang), and Changxing County (northern Zhejiang). Participants were initially recruited through official contacts at municipal health commissions and hospital administrators, who distributed the online questionnaire link to eligible HCWs within their respective institutions. The target population comprised registered HCWs (doctors, nurses, medical technicians, and public health personnel) actively practicing in the selected cities' medical institutions during the survey period. To minimize selection bias, we implemented quota sampling within each city, aligning the sample distribution with the relative proportions of different HCW groups in Zhejiang Province as reported in official provincial health statistics (25). This approach resulted in a sample in which physicians and nurses collectively constituted the largest proportion, with public health personnel and medical technicians representing a smaller (collectively accounting for ~15%−20% of the target population).

### 2.2 Survey development and validation

#### 2.2.1 Item generation

The two-phase questionnaires were developed based on relevant Chinese official policies [the 2022 Second Booster Dose Implementation Plan (26) and the 2023 Vaccination Work Plan (27)]. The questionnaire collected basic demographic, professional, vaccination history, and vaccination status of HCWs' household members (children and elderly) data. In addition, specific survey items measuring key constructs (e.g., risk perception, vaccine beliefs) were informed by previously published surveys on HCW vaccination. Risk perception was measured using a 0–10 numeric rating scale (NRS) (28). Specifically, where 0 represented the most negative response (e.g., “no risk at all” for risk perception; “complete distrust” for trust), 10 indicated the most positive response (e.g., “extremely high risk”; “complete trust”). The section investigating reasons for not receiving the booster dose was structured around key constructs of the Health Belief Model (HBM) (29); agreement with these statements was measured on a 5-point Likert scale. Specifically, unvaccinated respondents were presented with statements reflecting perceived barriers (e.g., concerns about safety, adverse reactions, and lack of recommendation), perceived benefits (reverse-scored item on vaccine effectiveness), and perceived susceptibility (reverse-scored item on personal infection risk). The detailed mapping of specific items is provided in Supplementary Table 1.

#### 2.2.2 Questionnaire validation and reliability

The questionnaire was reviewed by a panel of three independent experts from the Zhejiang Provincial Center for Disease Control and Prevention (ZJCDC), specifically from the Department of Infectious Disease Prevention and Control. The experts rated each item on its relevance and clarity using a 4-point scale. The Scale-level Content Validity Index (S-CVI) was calculated, achieving an excellent value of 0.91, indicating high expert consensus on the appropriateness of the content (34). The questionnaire was pilot-tested with a sample of 50 HCWs who were not included in the main study. The pilot test assessed the questionnaire's clarity, comprehensibility, and average completion time. Internal consistency reliability for the multi-item scales was evaluated using Cronbach's alpha, producing a value of α > 0.75 that indicates acceptable internal consistency.

#### 2.2.3 Questionnaire hosting and distribution

The online questionnaire was hosted on the Wenjuanxing platform (https://www.wjx.cn), a widely used online survey tool in China comparable to Qualtrics or SurveyMonkey. Survey links were disseminated to participants primarily through the popular instant messaging app WeChat. After clicking the link, participants were presented with the online questionnaire. The first item served as the informed consent; only those who selected “agree” could proceed to the subsequent questions. All responses were anonymous and self-administered.

### 2.3 Statistical analyses

#### 2.3.1 Data preprocessing

After data export, preliminary screening was conducted to exclude responses from individuals who declined participation or whose answers demonstrated logical inconsistencies. Missingness in this study originated primarily from questionnaire skip logic (where subsequent questions were hidden based on prior responses). These programmatically missing responses were excluded from the specific analyses.

#### 2.3.2 Variable definition and handling

The primary outcome variable was participants' willingness to be vaccinated, which was operationalized as a binary variable. Respondents who answered “Yes” were classified as the “Willing” group, while those who selected “Unsure” or “No” were classified as the “Unwilling” group. This categorization was applied separately to the 2022 (fourth dose) and 2023 (annual vaccination) datasets. Continuous variables were recategorized to simplify the interpretation of the logistic regression model coefficients. The risk perception score (0–10 scale) was transformed into a three-level ordinal variable: Mild (0–3), corresponding to below-median perception; Medium (4–6), approximating the median range; and Serious (7–10), representing the upper tertile.

#### 2.3.3 Software and analytical strategy

All statistical analyses were performed using R software (v4.4.2). We compared demographic and clinical characteristics between the Willing and Unwilling groups using Chi-squared or Fisher's exact tests for categorical variables. Variables with a *p*-value < 0.05 in univariate analysis were included in a multivariable binary logistic regression model to identify factors independently associated with vaccination willingness. Variable selection was performed using a bidirectional stepwise algorithm based on the Akaike Information Criterion (AIC). Multicollinearity was assessed using variance inflation factors (VIF), and all final predictors had a VIF < 3, indicating no substantial collinearity.

### 2.4 Ethics statement

This study was approved by the Ethics Review Committee of the Zhejiang Provincial Center for Disease Control and Prevention (Approval No.: 2022-021-01) and was conducted in accordance with the principles of the Declaration of Helsinki.

## 3 Results

### 3.1 Participant characteristics and COVID-19 vaccination status

From July to September 2022, 822 questionnaires were collected across five prefecture-level cities, with 820 valid responses. Participants were predominantly female (71.34%, 585/820) and young to middle-aged (18-35 years, 45.73%, 375/820). Geographically, 63.66% (522/820) resided in urban areas, whereas 36.34% (298/820) were from rural or peri-urban areas. Most participants held a university degree (including college, 91.71%, 752/820); clinical medicine (32.93%, 270/820) and nursing (32.56%, 267/820) were the most common specialties. A majority of participants had at least 10 years of professional experience (58.90%, 483/820).

From November 2023 to January 2024, 840 questionnaires were collected, with 837 valid responses. Participants were again predominantly female (73.48%, 615/837), and most were aged 18–35 years (46.59%, 390/837). The majority held a university degree (87.34%, 737/837); clinical medicine (40.26%, 337/837) and nursing (32.14%, 269/837) remained the top specialties. More than half of the participants had over 10 years of professional experience (57.83%, 484/837). The urban-rural distribution was nearly balanced (~50% each), as shown in Table 1.

**Table 1:** Participant characteristics of HCWs and the fourth dose of COVID-19 vaccination intentions (2022) & annual vaccination intentions (2023).

**Variables**	**Total (2022, *n* = 820)**	**Willingness to receive the fourth dose of COVID-19 vaccination**		**statistic**	***P***	**Total (2023, *n* = 837)**	**Willingness to vaccinate annually for COVID-19 vaccination**		**statistic**	***P***
		**Willing (*****n*** = **579)**	**Unwilling (*****n*** = **241)**				**Willing (*****n*** = **238)**	**Unwilling (*****n*** = **599)**		
**Gender**, ***n*****(%)**										
Male	235 (28.66)	187 (79.57)	48 (20.43)	χ^2^ = 12.76	**< 0.001**	222 (26.52)	76 (34.23)	146 (65.77)	χ^2^ = 4.99	**0.025**
Female	585 (71.34)	392 (67.01)	193 (32.99)			615 (73.48)	162 (26.34)	453 (73.66)		
**Age**, ***n*****(%)**										
18–35 years	375 (45.73)	259 (69.07)	116 (30.93)	χ^2^ = 3.19	0.364	390 (46.59)	104 (26.67)	286 (73.33)	χ^2^ = 9.12	**0.028**
36–45 years	264 (32.20)	184 (69.70)	80 (30.30)			256 (30.59)	73 (28.52)	183 (71.48)		
46–60 years	172 (20.98)	128 (74.42)	44 (25.58)			170 (20.31)	49 (28.82)	121 (71.18)		
>60 years	9 (1.10)	8 (88.89)	1 (11.11)			21 (2.51)	12 (57.14)	9 (42.86)		
**Residence**, ***n*****(%)**										
Urban area	522 (63.66)	368 (70.50)	154 (29.50)	χ^2^ = 0.01	0.926	417 (49.82)	123 (29.50)	294 (70.50)	χ^2^ = 0.46	0.498
Suburban/rural	298 (36.34)	211 (70.81)	87 (29.19)			420 (50.18)	115 (27.38)	305 (72.62)		
**Education**, ***n*****(%)**										
Senior secondary	53 (6.46)	38 (71.70)	15 (28.30)	χ^2^ = 1.95	0.377	94 (11.23)	39 (41.49)	55 (58.51)	χ^2^ = 9.48	**0.009**
University (including post-secondary)	752 (91.71)	528 (70.21)	224 (29.79)			731 (87.34)	197 (26.95)	534 (73.05)		
Master's degree or above	15 (1.83)	13 (86.67)	2 (13.33)			12 (1.43)	2 (16.67)	10 (83.33)		
**Medicine profession**, ***n*****(%)**										
Clinical Medicine	270 (32.93)	197 (72.96)	73 (27.04)	χ^2^ = 14.55	**0.006**	337 (40.26)	114 (33.83)	223 (66.17)	χ^2^ = 17.33	**0.002**
Preventive Medicine	129 (15.73)	90 (69.77)	39 (30.23)			91 (10.87)	21 (23.08)	70 (76.92)		
Inspection	51 (6.22)	46 (90.20)	5 (9.80)			36 (4.30)	9 (25.00)	27 (75.00)		
Nursing	267 (32.56)	173 (64.79)	94 (35.21)			269 (32.14)	56 (20.82)	213 (79.18)		
Non-medical	103 (12.56)	73 (70.87)	30 (29.13)			104 (12.43)	38 (36.54)	66 (63.46)		
**Seniority**, ***n*****(%)**										
≦3 years	94 (11.46)	70 (74.47)	24 (25.53)	χ^2^=0.97	0.616	110 (13.14)	26 (23.64)	84 (76.36)	χ^2^=1.54	0.463
4-10 years	243 (29.63)	173 (71.19)	70 (28.81)			243 (29.03)	69 (28.40)	174 (71.60)		
≧10 years	483 (58.90)	336 (69.57)	147 (30.43)			484 (57.83)	143 (29.55)	341 (70.45)		
**Primary series (typically 2 doses)**, ***n*****(%)**										
Vaccinated	817 (99.63)	579 (70.87)	238 (29.13)	-^a^	**0.025**	831 (99.28)	238 (28.64)	593 (71.36)	χ^2^ = 1.20	0.273
Unvaccinated	3 (0.37)	0 (0.00)	3 (100.00)			6 (0.72)	0 (0.00)	6 (100.00)		
**Booster dose**, ***n*****(%)**										
Vaccinated	799 (97.44)	579 (72.47)	220 (27.53)	χ^2^ = 51.78	**< 0.001**	777 (92.83)	232 (29.86)	545 (70.14)	χ^2^ = 10.79	**0.001**
Unvaccinated	21 (2.56)	0 (0.00)	21 (100.00)			60 (7.17)	6 (10.00)	54 (90.00)		

^a^Due to the small sample size, this group was tested using the Fisher exact. Other comparisons between groups were made using the χ^2^, Chi-square test.

In 2022, 99.63% of participants had completed the primary vaccination series (typically two doses), 97.44% had received a booster dose, and 70.61% (579/820) reported willingness to receive a fourth dose. In 2023, COVID-19 vaccine coverage rates remained high for both the primary series (99.28%) and booster doses (92.80%); the willingness for annual vaccination was 28.43% (238/837).

### 3.2 Risk perception of SARS-CoV-2

**Figure 1:**
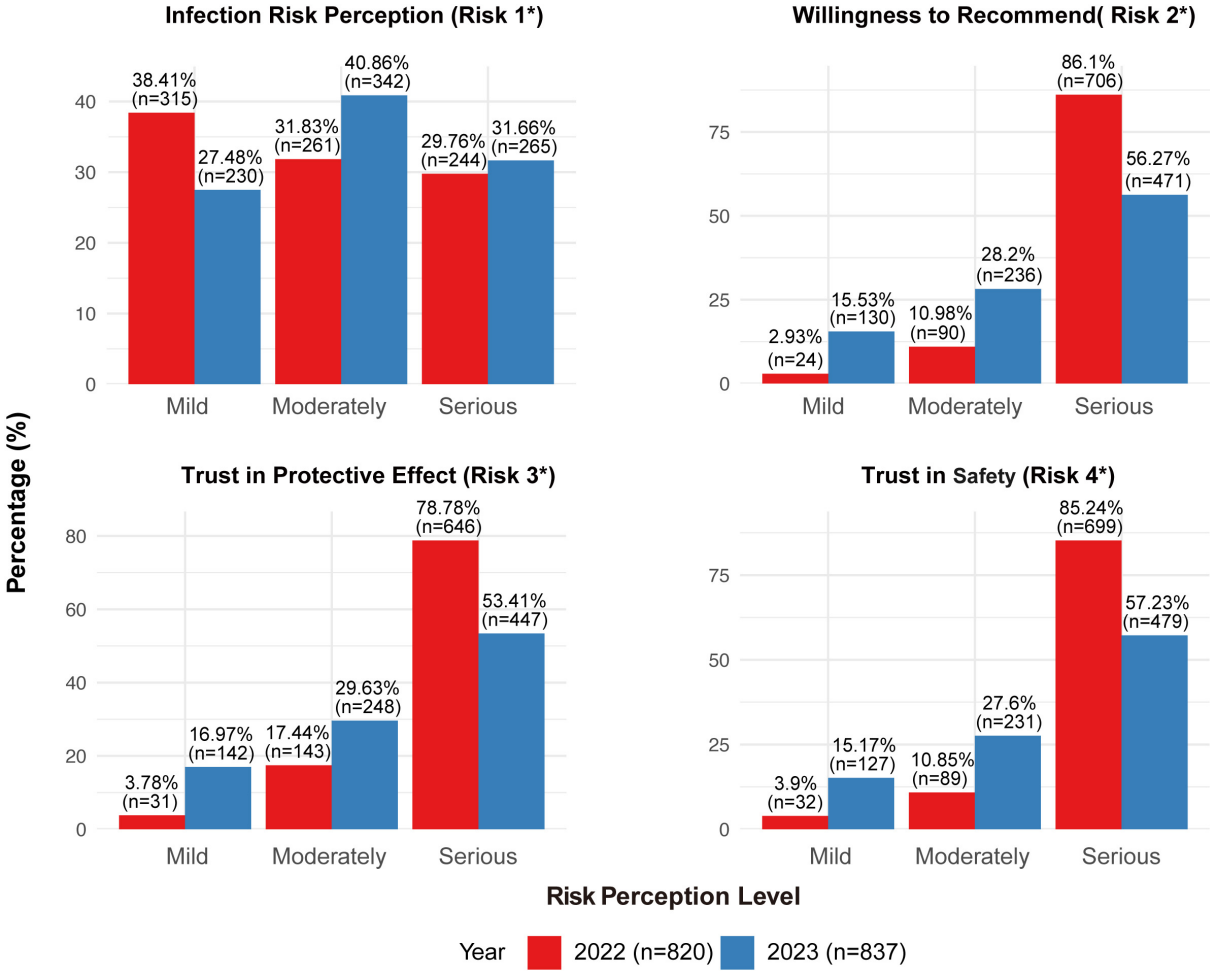
Characteristics of healthcare workers' perception of SARS-CoV-2 risk (2022–2023). Risk 1: What do you think your risk of contracting SARS-CoV-2 is? Risk 2: How likely are you to recommend the COVID-19 vaccination to someone who asks for your advice? Risk 3: How much do you trust the protective effect of COVID-19 vaccination? Risk 4: How much do you trust the safety of COVID-19 vaccination?

**Figure 2:**
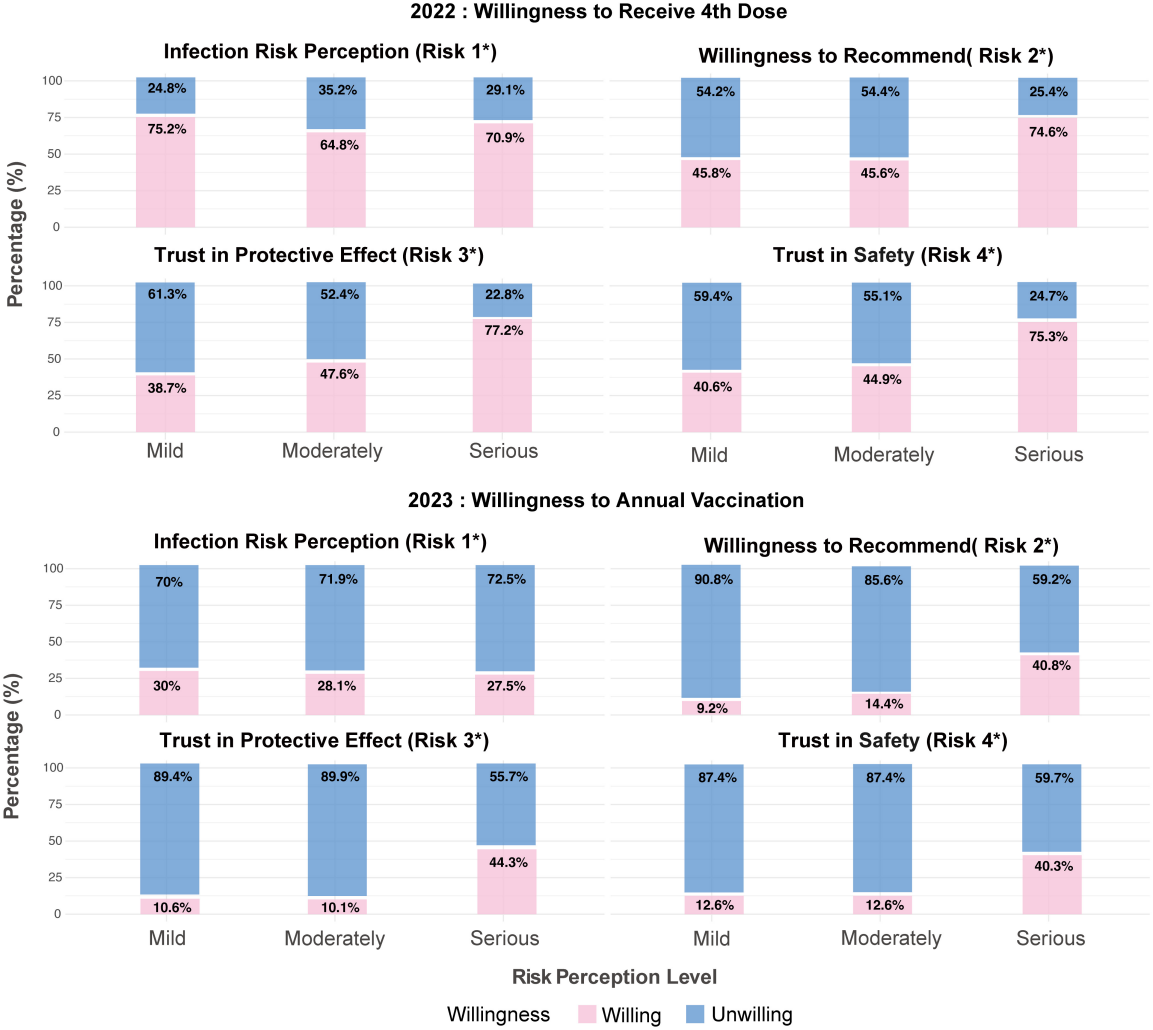
Distribution of vaccination willingness groups across risk perception levels. Risk 1: What do you think your risk of contracting SARS-CoV-2 is? Risk 2: How likely are you to recommend the COVID-19 vaccination to someone who asks for your advice? Risk 3: How much do you trust the protective effect of COVID-19 vaccination? Risk 4: How much do you trust the safety of COVID-19 vaccination?

Among 820 participants in 2022, most perceived their risk of SARS-CoV-2 infection as mild (38.41%, 315/820) or moderate (31.83%, 261/820). The majority of participants expressed strong confidence in vaccine safety (85.24%, 699/820) and protective efficacy (78.78%, 646/820); they reported a high willingness to recommend vaccination to eligible individuals seeking their advice (86.10%, 706/820). In the 2023 survey, a higher proportion of participants perceived their infection risk as moderate (40.86%, 342/837) or high (31.66%, 265/837) compared to 2022. However, confidence in vaccine safety (57.23%, 479/837), protective efficacy (53.41%, 447/837), and willingness to recommend vaccination (56.27%, 471/837) all declined (Figures 1, 2).

### 3.3 Multifactorial analysis of factors associated with vaccination

**Figure 3:**
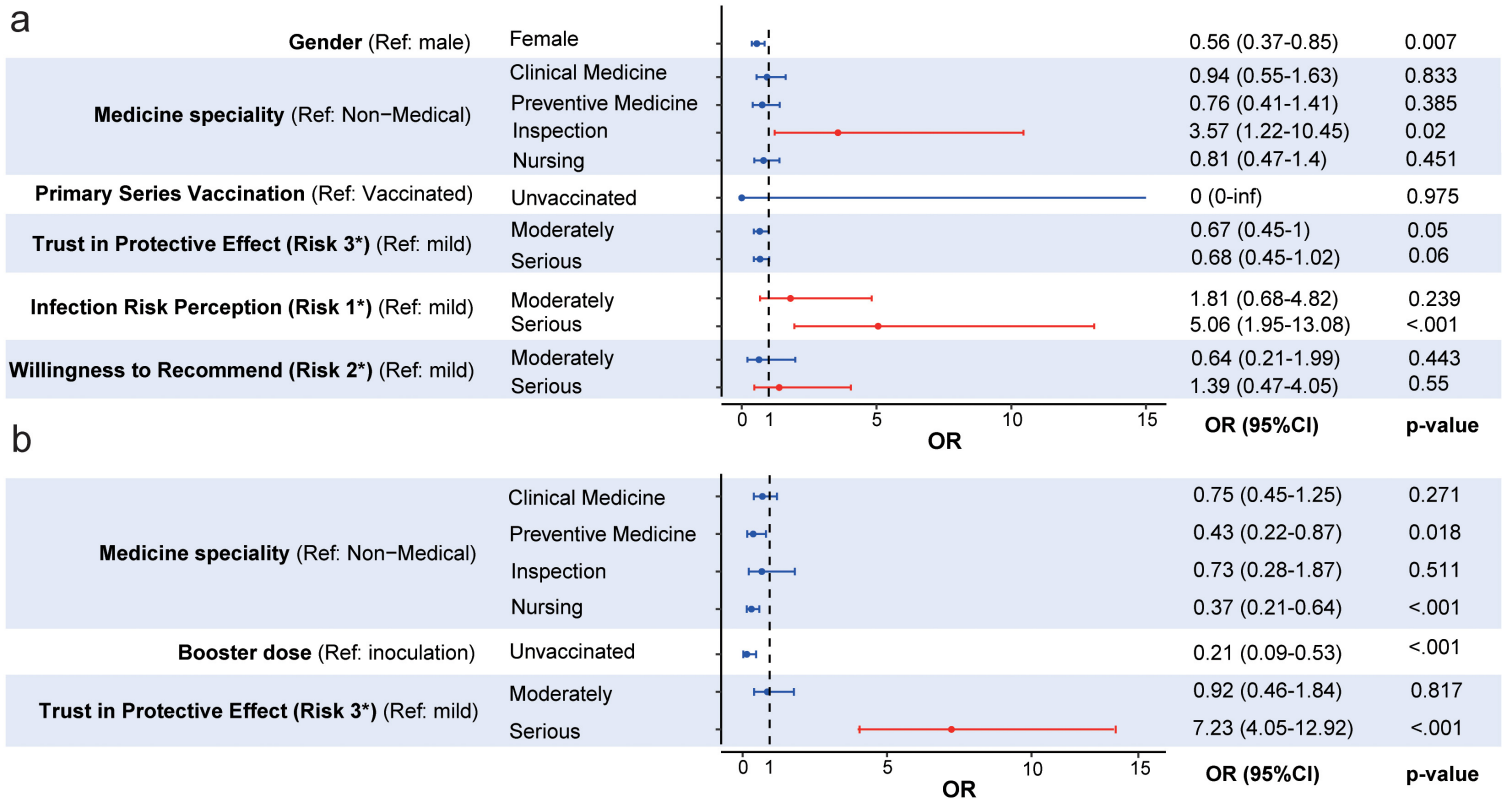
Multifactorial logistic regression analysis of willingness to receive the 4th dose of COVID-19 vaccination **(a)** and annual vaccination intention **(b)**. Risk 1: What do you think your risk of contracting SARS-CoV-2 is? Risk 2 :How likely are you to recommend the COVID-19 vaccination to someone who asks for your advice? Risk 3 :How much do you trust the protective effect of COVID-19 vaccination?

Univariate analysis (*p* < 0.05) identified gender, medical specialty, completion of prior immunization, and SARS-CoV-2 risk perception as factors associated with HCWs' willingness to receive the fourth dose of the COVID-19 vaccine in 2022. The 2023 survey revealed that gender, age, education level, medical specialty, completion of the booster dose, and SARS-CoV-2 risk perception were linked to willingness to undergo annual vaccination in this population (Table 1). A binary logistic regression analysis was performed using the statistically significant factors as independent variables and vaccination willingness as the dependent variable. The results showed that women had lower willingness to be vaccinated (OR = 0.56, 95% CI: 0.37–0.85). Relative to non-medical professionals, laboratory personnel showed higher willingness to receive the fourth dose (OR = 3.57, 95% CI: 1.22–10.45); participants with high confidence in vaccine efficacy were more willing (OR = 5.06, 95% CI: 1.95–13.08) (Figure 3a). Concerning annual COVID-19 vaccination, completion of prior vaccination and high confidence in protective efficacy remained significant predictors of willingness (OR = 7.23, 95% CI: 4.05–12.92) (Figure 3b).

### 3.4 Reasons for non-completion of the full COVID-19 vaccination series

In 2022, only 2.56% of participants had not received a booster dose, primarily citing the belief that the booster offered insufficient protection (50%). separately, the proportion of unboosted participants was 7.17% in the 2023 survey; “unsuitable for vaccination due to medical reasons” (50%) was the most common reason, followed by “no one advised me to get vaccinated” (33.33%). Most unvaccinated participants rejected views suggesting insufficient vaccine protection, serious adverse reactions, or unnecessary vaccination; the majority acknowledged a persistent risk of infection and agreed on the need for vaccination (Supplementary Figure 1).

### 3.5 HCWs' household vaccination recommendations

Vaccination coverage within HCWs' households was also examined. Analysis of the 2022 data revealed that households demonstrated higher completion rates for full vaccination among older members but lower acceptance of booster doses for children. Older household members had high completion rates for both the primary series (94.83%) and booster doses (86.02%). In contrast, booster vaccination rates for children were markedly lower (35.85%) than those for the primary series (85.13%) (Tables 2, 3).

**Table 2:** Status of families vaccinating their children against COVID-19 vaccination.

**Variables**	**Total (*n* = 491)**	**Whether the child receives the primary series (typically 2 doses) of COVID-19 vaccination**				**Whether the child receives a booster dose of COVID-19 vaccination**			
		**Vaccinated** **(*****n*** = **418)**	**Unvaccinated (*****n*** = **34)**	**Statistic**	***P***	**Vaccinated (*****n*** = **176)**	**Unvaccinated (*****n*** = **315)**	**Statistic**	***P***
**Child Age**, ***n*****(%)**									
≦3 years	57 (11.61)	4 (7.02)	53 (92.98)	χ^2^ = 312.09	**< 0.001**	3 (75.00)	54 (94.74)	χ^2^ = 27.98	**< 0.001**
3–12 years	311 (63.34)	293 (94.21)	18 (5.79)			118 (40.27)	193 (62.06)		
13–17 years	123 (25.05)	121 (98.37)	2 (1.63)			55 (45.45)	68 (55.28)		

**Table 3:** Status of families vaccinating the elderly against COVID-19 vaccination.

**Variables**	**Total (*n* = 658)**	**Whether the elder receive the primary series (typically 2 doses) of COVID-19 vaccination**				**Whether the elderly receive a booster dose of COVID-19 vaccination**			
		**vaccinated** **(*****n*** = **624)**	**unvaccinated (*****n*** = **34)**	**statistic**	***P***	**vaccinated** **(*****n*** = **566)**	**unvaccinated (*****n*** = **92)**	**statistic**	***P***
**Elder Age**, ***n*****(%)**									
60–80 years	592 (89.97)	565 (95.44)	27 (4.56)	χ^2^ = 3.28	0.070	522 (88.18)	70 (11.82)	χ^2^ = 22.84	**< 0.001**
>80 years	66 (10.03)	59 (89.39)	7 (10.61)			44 (66.67)	22 (33.33)		
**Presence of underlying disease**, ***n*****(%)**									
Yes	475 (72.19)	442 (93.05)	33 (6.95)	χ^2^ = 11.05	**< 0.001**	394 (82.95)	81 (17.05)	χ^2^ = 13.39	**< 0.001**
No	183 (27.81)	182 (99.45)	1 (0.55)			172 (93.99)	11 (6.01)		

## 4 Discussion

Phase 1 findings revealed high willingness and strong intent to recommend the fourth dose of COVID-19 vaccination among HCWs in Zhejiang Province. However, willingness was lower among female HCWs; laboratory testing personnel and individuals with high trust in vaccine safety and efficacy exhibited greater acceptance.

Multiple studies have identified gender, age, occupation, vaccination history, and trust as key factors influencing HCWs' willingness to receive COVID-19 vaccines (30–32). Most findings are consistent with the present results; for instance, women generally show greater hesitancy than men. However, some reports noted lower vaccine uptake among male HCWs (33), whereas others identified no significant gender differences in overall vaccine acceptance (34). Univariate analysis in the present study revealed that multiple dimensions of risk perception influenced vaccination willingness. HCWs with greater trust in the protective efficacy of COVID-19 vaccines were more likely to accept subsequent doses, in line with surveys on public willingness to receive booster doses in China (35).

Phase 2 findings indicated low willingness among HCWs to undergo annual COVID-19 vaccination. Willingness was low among nurses; laboratory testing personnel, individuals who had completed prior vaccination, and high-trust groups maintained greater acceptance.

Multiple studies have indicated that nurses exhibit lower willingness than physicians to receive COVID-19 vaccines (30, 36), although others have revealed higher willingness among female nurses specifically (37).

Taken together, our study captured the vaccination willingness characteristics of Chinese HCWs in two different contexts, these two characteristics were highly correlated with their respective trust levels and information environments.

The high vaccination willingness in 2022 was primarily associated with strong trust in vaccines and a heightened perception of risk among HCWs. Our study found that trust was a key factor influencing HCWs. Furthermore, survey data showed that, regardless of their vaccination willingness, 85.24% of participants highly trusted vaccine safety and 78.78% highly trusted vaccine efficacy at that time. This personal trust in vaccines was underpinned by a broader institutional trust; consistent recommendations from health authorities framed vaccination as a critical public health duty, which HCWs largely endorsed. Consequently, this foundation of trust, combined with a clear awareness of personal and occupational risk, served as the critical driver for vaccine uptake. The higher acceptance observed among laboratory personnel and other high-trust groups further confirms the positive role of scientific literacy and institutional trust in promoting vaccination (35). This culture of trust may have also facilitated HCWs' role as vaccination advocates within their families, thereby amplifying the broader public health impact of confident healthcare workers.

The survey results from 2023 present a distinct profile of attitudes compared to 2022, reflecting the changed context of widespread prior infection and established population immunity. While a higher proportion of HCWs in 2023 perceived their infection risk as moderate or high, this increased risk perception did not translate into stronger vaccination intent. Relative to 2022, the key motivational drivers were observed at lower levels: confidence in vaccine safety and protective efficacy, and willingness to recommend vaccination to others. Therefore, in the post-pandemic era, despite the persistent risk of infection, HCWs' perception of the need for vaccination underwent a shift. This change in attitude aligns with the characteristics of the pandemic's transition to a “new normal” phase (38). As public health priorities shifted from emergency response to routine management, the perceived urgency and attention accorded to vaccination among the public, including HCWs, naturally diminished (39). This shift is further contextualized by our finding that 99.28% of participants had completed a primary COVID-19 vaccination series and 92.80% had received booster doses by 2023, suggesting a background of collective immunity likely influenced the recalibrated perception of vaccination necessity. At the individual level, this translated to a tendency for those who had established an immune foundation through prior vaccination to be more inclined to maintain this protective status, a finding consistent with studies showing that prior vaccination experience positively influences subsequent willingness. Finally, as public health communication pivoted to focus on older and high-risk groups, proactive messaging toward HCWs became less intensive than in 2022. At the same time, the psychological state of HCWs evolved within the new context, with factors such as concerns about adverse reactions having a more pronounced impact on the willingness of female HCWs and nurses (40).

Building on our findings, tailored strategies can further enhance vaccination willingness and optimize routine immunization programs among HCWs. Communication should be specialized by profession: for instance, providing laboratory staff with precise technical data (e.g., neutralizing antibody titers, quality control metrics) can strengthen their informed support (41), whereas nurses may be more effectively engaged with evidence of clinical impact, such as reductions in patient mortality or nosocomial infections (42). These efforts would help sustain trust in vaccine safety and reinforce the understanding that endemic virus circulation necessitates ongoing protection. Furthermore, establishing system-level supports is essential for success (43). This could include implementing regular vaccine education that clearly outlines protection duration and efficacy, setting up support hotlines (particularly for female HCWs and nurses) to address questions, and creating structured feedback channels between frontline staff and policy makers. Such measures would facilitate the integration of vaccination into routine clinical practice beyond the pandemic phase (44).

## 5 Limitations

This study has several limitations. The two-phase independent survey design captured population-level attitudes at two critical junctures but does not permit analysis of individual-level changes over time. The exclusive use of self-report questionnaires risks recall and social desirability bias. The online voluntary sampling, while practical, may have attracted HCWs with stronger health interests, and the gender imbalance, though reflective of the nursing-dominated workforce, may affect the generalizability of findings to all HCW subgroups. As a study conducted in Zhejiang Province, its findings should be considered within the context of its specific regional socioeconomic and public health conditions. Finally, the questionnaire demonstrated good face validity and reliability, though future studies would benefit from more extensive validation of the psychological constructs.

## 6 Conclusion

This two-phase study delineates the characteristics of COVID-19 vaccine willingness among Chinese HCWs before and after the pandemic. High willingness in the late pandemic phase was closely associated with strong vaccine confidence and clear risk perception, whereas low willingness in the post-pandemic era correlated with shifts in trust levels, a weakening of risk perception, and a changed decision-making environment. Across both phases, trust in vaccines emerged as the most consistent and powerful driver of acceptance. These findings offer insights for strategies aimed at improving vaccination willingness. Sustaining confidence through transparent information and promoting positive perceptions of vaccination will be key to maintaining high acceptance levels among HCWs.

## Data Availability

The raw data supporting the conclusions of this article will be made available by the authors, without undue reservation.
