# Supplementary material for: Exploring longitudinal trends and multifactorial correlations of COVID-19 vaccination willingness among healthcare workers in China: a two-phase cross-sectional study before and after the 2023 phase of COVID-19 pandemic
Source: Front Public Health. 2025 Nov 10;13:1699531. doi: 10.3389/fpubh.2025.1699531 (PMC12640930; doi:10.3389/fpubh.2025.1699531)
Supplement: Supplementary file 1 [file Table_1.docx]

**Supplementary Table 1: Mapping of Survey Items for Non-Vaccination onto Health Belief Model (HBM) Constructs**

**The 2023 survey is used as the example here because it included additional items (No.10(6) and No.13(8) regarding prior infection) not present in the 2022 questionnaire, providing a more comprehensive mapping. All survey items from the 2023 questionnaire are listed below.**

| **Vaccination Stage** | **HBM Construct and Conceptual Definition** | **Survey Item Number & Description** |
| --- | --- | --- |
| Reasons for not receiving the **primary series** | **Perceived Barriers** *Beliefs about the tangible and psychological costs of the advised action.* | 10(1). I have health conditions that make me unsuitable for the COVID-19 vaccine. 10(3). The COVID-19 vaccine can cause serious adverse reactions. 10(5). No one informed me that I should get vaccinated. |
|  | **Perceived Benefits** *Belief in the efficacy of the advised action to reduce risk or seriousness of impact.* | 10(2). The COVID-19 vaccine is not effective. (Reverse-scored) |
|  | **Perceived Susceptibility** *Subjective assessment of risk of developing a condition.* | 10(4). I will not get infected with COVID-19. (Reverse-scored) |
|  | **Perceived Susceptibility/Benefits** *Overlap in assessing personal risk and the action's relevance.* | 10(6). I have already been infected, so vaccination is unnecessary. |
| Reasons for not receiving the **Booster Dose** | **Perceived Barriers** *Beliefs about the tangible and psychological costs of the advised action.* | 13(2). I have health conditions that make me unsuitable for the COVID-19 booster vaccine. 13(4). The COVID-19 booster shot can cause more severe adverse reactions. 13(5). The adverse reactions I experienced from previous doses were severe... 13(7). No one informed me that I should get the booster shot. |
|  | **Perceived Benefits** *Belief in the efficacy of the advised action to reduce risk or seriousness of impact.* | 13(3). The COVID-19 vaccine is not effective. (Reverse-scored) |
|  | **Perceived Susceptibility** *Subjective assessment of risk of developing a condition.* | 13(6). I will not get infected with COVID-19. (Reverse-scored) |
|  | **Cues to Action** *Strategies to activate readiness or promote compliance.* | 13(1). It is not yet time for me to receive the booster shot. |
|  | **Perceived Susceptibility/Benefits** *Overlap in assessing personal risk and the action's relevance.* | 13(8). I have already been infected, so a booster is unnecessary. |
