# Supplementary material for: Exploring longitudinal trends and multifactorial correlations of COVID-19 vaccination willingness among healthcare workers in China: a two-phase cross-sectional study before and after the 2023 phase of COVID-19 pandemic
Source: Front Public Health. 2025 Nov 10;13:1699531. doi: 10.3389/fpubh.2025.1699531 (PMC12640930; doi:10.3389/fpubh.2025.1699531)
Supplement: Supplementary file 2 [file Table_2.docx]

COVID-19 Vaccine Booster Shot Willingness Survey for HCWs, 2022

To understand the public's willingness to receive future COVID-19 vaccines, we sincerely invite you to participate in this survey. Your participation will contribute to COVID-19 vaccination efforts and subsequent epidemic prevention and control. This survey is anonymous and does not involve personal privacy. Your responses will be coded for analysis. Please feel free to fill it out.

Thank you very much for taking the time to complete this questionnaire!

1. Do you agree to participate in this survey?

* Yes

* No

2. What is your gender?

* Male

* Female

3. What is your age?

* 18-35 years old

* 36-45 years old

* 46-60 years old

* >60 years old

4. What is your current area of residence?

* Urban area

* Suburban/Rural area

5. What is your highest level of education?

* High school (or technical secondary school)

* University (or junior college)

* Master's degree or above

6. What is your medical background?

* Clinical Medicine

* Preventive Medicine

* Laboratory Science

* Nursing

* Non-medical background

7. How many years have you worked in your current position?

* Less than or equal to 3 years

* 4-10 years

* More than 10 years

8. Risk Perception and Trust (Higher score indicates higher degree)

(1) How great do you think your risk of contracting COVID-19 is?

(0=No risk, 10=Very high risk)

`0 1 2 3 4 5 6 7 8 9 10`

(2) What is your level of trust in the safety of China's COVID-19 vaccines?

(0=No trust, 10=Complete trust)

`0 1 2 3 4 5 6 7 8 9 10`

(3) What is your level of trust in the protective effect of China's COVID-19 vaccines?

(0=No trust, 10=Complete trust)

`0 1 2 3 4 5 6 7 8 9 10`

(4) To what degree would you recommend the COVID-19 vaccine to someone consulting you?

(0=Would not recommend, 10=Highly recommend)

`0 1 2 3 4 5 6 7 8 9 10`

9. Have you been vaccinated with the COVID-19 vaccine (primary series)?(Select No to jump to 12)

* Yes

* No

10. Have you received the COVID-19 vaccine booster shot?(Select No to jump to 13)

* Yes

* No

11. If a 4th dose is recommended by the national or provincial authorities, would you be willing to get it?

* Yes

* No

* Uncertain

12. If you have NOT been vaccinated, what are the reasons?(Jump to 14 upon completion)

(Please indicate your level of agreement for each statement: 1=Strongly Disagree, 2=Disagree, 3=Neutral/Don't Know, 4=Agree, 5=Strongly Agree)

| Statement | 1 | 2 | 3 | 4 | 5 |
| --- | --- | --- | --- | --- | --- |
| (1) I have health conditions that make me unsuitable for the COVID-19 vaccine. | ○ | ○ | ○ | ○ | ○ |
| (2) The COVID-19 vaccine is not effective. | ○ | ○ | ○ | ○ | ○ |
| (3) The COVID-19 vaccine can cause serious adverse reactions. | ○ | ○ | ○ | ○ | ○ |
| (4) I will not get infected with COVID-19. | ○ | ○ | ○ | ○ | ○ |
| (5) No one informed me that I should get vaccinated. | ○ | ○ | ○ | ○ | ○ |

13. If you have NOT received the booster shot, what are the reasons?(Jump to 15 upon completion)

(Please indicate your level of agreement for each statement: 1=Strongly Disagree, 2=Disagree, 3=Neutral/Don't Know, 4=Agree, 5=Strongly Agree)

| Statement | 1 | 2 | 3 | 4 | 5 |
| --- | --- | --- | --- | --- | --- |
| (1) It is not yet time for me to receive the booster shot. | ○ | ○ | ○ | ○ | ○ |
| (2) I have health conditions that make me unsuitable for the COVID-19 booster vaccine. | ○ | ○ | ○ | ○ | ○ |
| (3) The COVID-19 vaccine is not effective. | ○ | ○ | ○ | ○ | ○ |
| (4) The COVID-19 booster shot can cause more severe adverse reactions. | ○ | ○ | ○ | ○ | ○ |
| (5) The adverse reactions I experienced from previous COVID-19 vaccine doses were severe enough to make me unwilling to get the booster. | ○ | ○ | ○ | ○ | ○ |
| (6) I will not get infected with COVID-19. | ○ | ○ | ○ | ○ | ○ |
| (7) No one informed me that I should get the booster shot. | ○ | ○ | ○ | ○ | ○ |

14. Are you willing to receive the COVID-19 vaccine in the future?

* Yes

* No

* Uncertain

15. Are you willing to receive a COVID-19 vaccine booster shot?

* Yes

* No

* Uncertain

16. Do you have a child/children aged 3-17 in your household? (Please answer the following questions for ONE selected child)

* No

* Yes, aged 3-12 years old

* Yes, aged 13-17 years old

* (Note: Option for under 3 years old was in original but not applicable for vaccine Qs)

17. Has this child received the COVID-19 vaccine (primary series)?

* Yes

* No

18. Has this child received the COVID-19 vaccine booster shot?

* Yes

* No

19. Do you have an elderly person/people aged 60 or above in your household? (Please answer the following questions for ONE selected elderly person)

* No

* Yes, aged 60-80 years old

* Yes, aged over 80 years old

20. Does this elderly person have any of the following underlying diseases? (Select at least one; currently selected: 0)

* No

* Yes

21. Has this elderly person received the COVID-19 vaccine (primary series)?

* Yes

* No

22. Has this elderly person received the COVID-19 vaccine booster shot?

* Yes

* No

Thank you very much for your suppor!

# 2022年医疗卫生人员新冠疫苗加强针接种意愿调查

为了解公众对未来新冠病毒疫苗的接种意愿，诚挚邀请您参与本问卷调查。您的参与将有助于新冠疫苗接种和后续新冠疫情防控工作。本次调查采取匿名形式，不涉及个人隐私，您的回答也将采用编码处理，请您放心填写。

衷心感谢您抽出宝贵时间填写这份问卷！

1. 请问您是否同意参加这次问卷调查？

○ 是

○ 否

2. 请问您的性别是？

○ 男

○ 女

3. 请问您的年龄是？

○ 18-35周岁

○ 36-45周岁

○ 46-60周岁

○ >60周岁

4. 请问您目前居住地是市区还是城郊/乡村？

○ 市区

○ 城郊/乡村

5. 请问您的最高教育水平是？

○ 高中（含中专）

○ 大学（含大专）

○ 硕士及以上

6. 请问您的医学背景？

○ 临床医学专业

○ 预防医学专业

○ 检验专业

○ 护理专业

○ 非医学专业

7. 请问您从事本岗位多少年了？

○ 不超过3年

○ 4-10年

○ 10年以上

8. 风险感知和信任（分值越大，程度越高）

(1) 您觉得自己感染新冠病毒的风险有多大？
0 — 1 — 2 — 3 — 4 — 5 — 6 — 7 — 8 — 9 — 10

(2) 您对我国新冠疫苗安全性的信任程度？
0 — 1 — 2 — 3 — 4 — 5 — 6 — 7 — 8 — 9 — 10

(3) 您对我国新冠疫苗保护效果的信任程度？
0 — 1 — 2 — 3 — 4 — 5 — 6 — 7 — 8 — 9 — 10

(4) 您对向您咨询的且可接种新冠疫苗人的推荐程度？
0 — 1 — 2 — 3 — 4 — 5 — 6 — 7 — 8 — 9 — 10

9. 请问您是否接种了新冠疫苗（基础针）？（选择否跳转12）

○ 是

○ 否

10. 请问您是否接种了新冠疫苗（加强针）？（选择否跳转13）

○ 是

○ 否

11. 如果国家或省里推荐接种第4针，您是否愿意接种？

○ 愿意

○ 不愿意

○ 不确定

12. 请问您未接种新冠疫苗的原因？（完成后跳转14）
（每题均为5个选项，数值表示：1.非常不赞同 2.不赞同 3.中立/不知道 4.赞同 5.非常赞同）

| 题目 | 1 | 2 | 3 | 4 | 5 |
| --- | --- | --- | --- | --- | --- |
| (1) 因为身体原因不适合接种新冠疫苗 | ○ | ○ | ○ | ○ | ○ |
| (2) 新冠疫苗不能够起到保护作用 | ○ | ○ | ○ | ○ | ○ |
| (3) 新冠疫苗会有严重的不良反应 | ○ | ○ | ○ | ○ | ○ |
| (4) 自己不会感染新冠病毒 | ○ | ○ | ○ | ○ | ○ |
| (5) 没有人告知要去接种新冠疫苗 | ○ | ○ | ○ | ○ | ○ |

13. 请问您未接种新冠疫苗（加强针）的原因？（完成后跳转15）
（每题均为5个选项，数值表示：1.非常不赞同 2.不赞同 3.中立/不知道 4.赞同 5.非常赞同）

| 题目 | 1 | 2 | 3 | 4 | 5 |
| --- | --- | --- | --- | --- | --- |
| (1) 未到加强免疫的接种时间 | ○ | ○ | ○ | ○ | ○ |
| (2) 因为身体原因不适合接种新冠疫苗加强针 | ○ | ○ | ○ | ○ | ○ |
| (3) 新冠疫苗加强针不能够起到保护作用 | ○ | ○ | ○ | ○ | ○ |
| (4) 新冠疫苗加强针会有更严重的不良反应 | ○ | ○ | ○ | ○ | ○ |
| (5) 既往接种新冠疫苗出现的不良反应严重程度让我不愿意接种加强针 | ○ | ○ | ○ | ○ | ○ |
| (6) 自己不会感染新冠病毒 | ○ | ○ | ○ | ○ | ○ |
| (7) 没有人告知要去接种新冠疫苗加强针 | ○ | ○ | ○ | ○ | ○ |

14. 请问您后续是否愿意接种新冠疫苗？

○ 愿意

○ 不愿意

○ 不确定

15. 请问您是否愿意接种新冠疫苗加强针？

○ 是

○ 否

○ 不确定

16. 您家中是否有3-17岁孩子？（选择家中其中一个孩子回答下述问题）

○ 无

○ 3周岁以下

○ 3周岁-12周岁

○ 13周岁-17周岁

17. 请问她/他是否接种了新冠疫苗？

○ 是

○ 否

18. 请问她/他是否接种了新冠疫苗加强针？

○ 是

○ 否

19. 您家中是否有60岁及以上的老人？（选择家中其中一个老人回答下述问题）

○ 无

○ 60-80周岁

○ >80周岁

20. 他/她是否有基础性疾病？

○ 无

○ 有

21. 请问她/他是否接种了新冠疫苗？

○ 是

○ 否

22. 请问她/他是否接种了新冠疫苗加强针？

○ 是

○ 否

非常感谢您的支持！

COVID-19 Vaccine Booster Shot Willingness Survey for HCWs, 2023

To understand the public's willingness to receive future COVID-19 vaccines, we sincerely invite you to participate in this survey. Your participation will contribute to COVID-19 vaccination efforts and subsequent epidemic prevention and control. This survey is anonymous and does not involve personal privacy. Your responses will be coded for analysis. Please feel free to fill it out.

Thank you very much for taking the time to complete this questionnaire!

1. Do you agree to participate in this survey?

* Yes

* No

2. What is your gender?

* Male

* Female

3. What is your age?

* 18-35 years old

* 36-45 years old

* 46-60 years old

* >60 years old

4. What is your current area of residence?

* Urban area

* Suburban/Rural area

5. What is your highest level of education?

* High school (or technical secondary school)

* University (or junior college)

* Master's degree or above

6. What is your medical background?

* Clinical Medicine

* Preventive Medicine

* Laboratory Science

* Nursing

* Non-medical background

7. How many years have you worked in your current position?

* Less than or equal to 3 years

* 4-10 years

* More than 10 years

8. Risk Perception and Trust (Higher score indicates higher degree)

(1) How great do you think your risk of subsequent COVID-19 infection is?

(0=No risk, 10=Very high risk)

`0 — 1 — 2 — 3 — 4 — 5 — 6 — 7 — 8 — 9 — 10`

(2) What is your level of trust in the safety of China's COVID-19 vaccines?

(0=No trust, 10=Complete trust)

`0 — 1 — 2 — 3 — 4 — 5 — 6 — 7 — 8 — 9 — 10`

(3) What is your level of trust in the protective effect of China's COVID-19 vaccines?

(0=No trust, 10=Complete trust)

`0 — 1 — 2 — 3 — 4 — 5 — 6 — 7 — 8 — 9 — 10`

(4) To what degree would you recommend the COVID-19 vaccine to someone consulting you?

(0=Would not recommend, 10=Highly recommend)

`0 — 1 — 2 — 3 — 4 — 5 — 6 — 7 — 8 — 9 — 10`

1. Have you been vaccinated with the COVID-19 vaccine (primary series)? (Select Yes to jump to 12)

* Yes

* No

1. If you have NOT been vaccinated, what are the reasons?

(Please indicate your level of agreement: 1=Strongly Disagree, 2=Disagree, 3=Neutral/Don't Know, 4=Agree, 5=Strongly Agree)

| Statement | 1 | 2 | 3 | 4 | 5 |
| --- | --- | --- | --- | --- | --- |
| (1) I have health conditions that make me unsuitable for the COVID-19 vaccine. | ○ | ○ | ○ | ○ | ○ |
| (2) The COVID-19 vaccine is not effective. | ○ | ○ | ○ | ○ | ○ |
| (3) The COVID-19 vaccine can cause serious adverse reactions. | ○ | ○ | ○ | ○ | ○ |
| (4) I will not get infected with COVID-19. | ○ | ○ | ○ | ○ | ○ |
| (5) No one informed me that I should get vaccinated. | ○ | ○ | ○ | ○ | ○ |
| (6) I have already been infected, so vaccination is unnecessary. | ○ | ○ | ○ | ○ | ○ |

11. Are you willing to receive the COVID-19 vaccine in the future?

* Yes

* No

* Unsure

12. Have you received the 3rd COVID-19 vaccine booster shot? (Select Yes to jump to 14)

* Yes

* No

13. If you have NOT received the 3rd booster shot, what are the reasons?

(Please indicate your level of agreement: 1=Strongly Disagree, 2=Disagree, 3=Neutral/Don't Know, 4=Agree, 5=Strongly Agree)

| Statement | 1 | 2 | 3 | 4 | 5 |
| --- | --- | --- | --- | --- | --- |
| (1) It is not yet time for me to receive the booster shot. | ○ | ○ | ○ | ○ | ○ |
| (2) I have health conditions that make me unsuitable for the COVID-19 booster vaccine. | ○ | ○ | ○ | ○ | ○ |
| (3) The COVID-19 vaccine is not effective. | ○ | ○ | ○ | ○ | ○ |
| (4) The COVID-19 booster shot can cause more severe adverse reactions. | ○ | ○ | ○ | ○ | ○ |
| (5) The adverse reactions I experienced from previous COVID-19 vaccine doses were severe enough to make me unwilling to get the booster. | ○ | ○ | ○ | ○ | ○ |
| (6) I will not get infected with COVID-19. | ○ | ○ | ○ | ○ | ○ |
| (7) No one informed me that I should get the booster shot. | ○ | ○ | ○ | ○ | ○ |
| (8) I have already been infected, so a booster is unnecessary. | ○ | ○ | ○ | ○ | ○ |

14. Are you willing to receive an annual COVID-19 vaccine?

* Yes

* No

* Unsure

15. Do you have a child/children aged 0-17 in your household? (Please answer for ONE selected child)

* No

* Yes, under 3 years old

* Yes, aged 3-12 years old

* Yes, aged 13-17 years old

16. Has this child received the COVID-19 vaccine (primary series)?

* Yes

* No

17. Has this child received the COVID-19 vaccine booster shot?

* Yes

* No

18. Do you have an elderly person/people aged 60 or above in your household? (Please answer for ONE selected elderly person)

* No

* Yes, aged 60-80 years old

* Yes, aged over 80 years old

1. Does this elderly person have any of the following underlying diseases?

* No

* Yes

20. Has this elderly person received the COVID-19 vaccine (primary series)?

* Yes

* No

21. Has this elderly person received the COVID-19 vaccine booster shot?

* Yes

* No

Thank you very much for your support

# 2023年医疗卫生人员新冠疫苗加强针接种意愿调查

为了解公众对未来新冠病毒疫苗的接种意愿，诚挚邀请您参与本问卷调查。您的参与将有助于新冠疫苗接种和后续新冠疫情防控工作。本次调查采取匿名形式，不涉及个人隐私，您的回答也将采用编码处理，请您放心填写。

衷心感谢您抽出宝贵时间填写这份问卷！

1. 请问您是否同意参加这次问卷调查？

○ 是

○ 否

2. 请问您的性别是？

○ 男

○ 女

3. 请问您的年龄是？

○ 18-35周岁

○ 36-45周岁

○ 46-60周岁

○ >60周岁

4. 请问您目前居住地是市区还是城郊/乡村？

○ 市区

○ 城郊/乡村

5. 请问您的最高教育水平是？

○ 高中（含中专）

○ 大学（含大专）

○ 硕士及以上

6. 请问您的医学背景？

○ 临床医学专业

○ 预防医学专业

○ 检验专业

○ 护理专业

○ 非医学专业

7. 请问您从事本岗位多少年了？

○ 不超过3年

○ 4-10年

○ 10年以上

8. 风险感知和信任（分值越大，程度越高）

(1) 您觉得自己后续感染新冠病毒的风险有多大？
0 — 1 — 2 — 3 — 4 — 5 — 6 — 7 — 8 — 9 — 10

(2) 您对我国新冠疫苗安全性的信任程度？
0 — 1 — 2 — 3 — 4 — 5 — 6 — 7 — 8 — 9 — 10

(3) 您对我国新冠疫苗保护效果的信任程度？
0 — 1 — 2 — 3 — 4 — 5 — 6 — 7 — 8 — 9 — 10

(4) 您对向您咨询的且可接种新冠疫苗人的推荐程度？
0 — 1 — 2 — 3 — 4 — 5 — 6 — 7 — 8 — 9 — 10

9. 请问您是否接种了新冠疫苗（基础针）？（选择是跳转12）

○ 是

○ 否

10. 请问您未接种新冠疫苗的原因？
（每题均为5个选项，数值表示：1.非常不赞同 2.不赞同 3.中立/不知道 4.赞同 5.非常赞同）

| 题目 | 1 | 2 | 3 | 4 | 5 |
| --- | --- | --- | --- | --- | --- |
| (1) 因为身体原因不适合接种新冠疫苗 | ○ | ○ | ○ | ○ | ○ |
| (2) 新冠疫苗不能够起到保护作用 | ○ | ○ | ○ | ○ | ○ |
| (3) 新冠疫苗会有严重的不良反应 | ○ | ○ | ○ | ○ | ○ |
| (4) 自己不会感染新冠病毒 | ○ | ○ | ○ | ○ | ○ |
| (5) 没有人告知要去接种新冠疫苗 | ○ | ○ | ○ | ○ | ○ |
| (6) 已经感染过新冠，没必要接种疫苗 | ○ | ○ | ○ | ○ | ○ |

11. 请问您后续是否愿意接种新冠疫苗？

○ 愿意

○ 不愿意

○ 不确定

12. 请问您是否接种了新冠疫苗（第3针加强针）？（选择是跳转14）

○ 是

○ 否

13. 请问您未接种新冠疫苗（第3针加强针）的原因？
（每题均为5个选项，数值表示：1.非常不赞同 2.不赞同 3.中立/不知道 4.赞同 5.非常赞同）

| 题目 | 1 | 2 | 3 | 4 | 5 |
| --- | --- | --- | --- | --- | --- |
| (1) 未到加强免疫的接种时间 | ○ | ○ | ○ | ○ | ○ |
| (2) 因为身体原因不适合接种新冠疫苗加强针 | ○ | ○ | ○ | ○ | ○ |
| (3) 新冠疫苗加强针不能够起到保护作用 | ○ | ○ | ○ | ○ | ○ |
| (4) 新冠疫苗加强针会有更严重的不良反应 | ○ | ○ | ○ | ○ | ○ |
| (5) 既往接种新冠疫苗出现的不良反应严重程度让我不愿意接种加强针 | ○ | ○ | ○ | ○ | ○ |
| (6) 自己不会感染新冠病毒 | ○ | ○ | ○ | ○ | ○ |
| (7) 没有人告知要去接种新冠疫苗加强针 | ○ | ○ | ○ | ○ | ○ |
| (8) 已经感染过新冠了，没必要再接种疫苗 | ○ | ○ | ○ | ○ | ○ |

14. 请问您是否愿意每年接种新冠疫苗？

○ 愿意

○ 不愿意

○ 不确定

15. 您家中是否有0-17岁孩子？（选择家中其中一个孩子回答下述问题）

○ 无

○ 3周岁以下

○ 3周岁-12周岁

○ 13周岁-17周岁

16. 请问她/他是否接种了新冠疫苗？

○ 是

○ 否

17. 请问她/他是否接种了新冠疫苗加强针？

○ 是

○ 否

18. 您家中是否有60岁及以上的老人？（选择家中其中一个老人回答下述问题）

○ 无

○ 60-80周岁

○ >80周岁

19. 他/她是否有基础性疾病？

○ 无

○ 有

20. 请问她/他是否接种了新冠疫苗？

○ 是

○ 否

21. 请问她/他是否接种了新冠疫苗加强针？

○ 是

○ 否

非常感谢您的支持！
