# Supplementary figures and images for: Exploring longitudinal trends and multifactorial correlations of COVID-19 vaccination willingness among healthcare workers in China: a two-phase cross-sectional study before and after the 2023 phase of COVID-19 pandemic
Source: Front Public Health. 2025 Nov 10;13:1699531. doi: 10.3389/fpubh.2025.1699531 (PMC12640930; doi:10.3389/fpubh.2025.1699531)

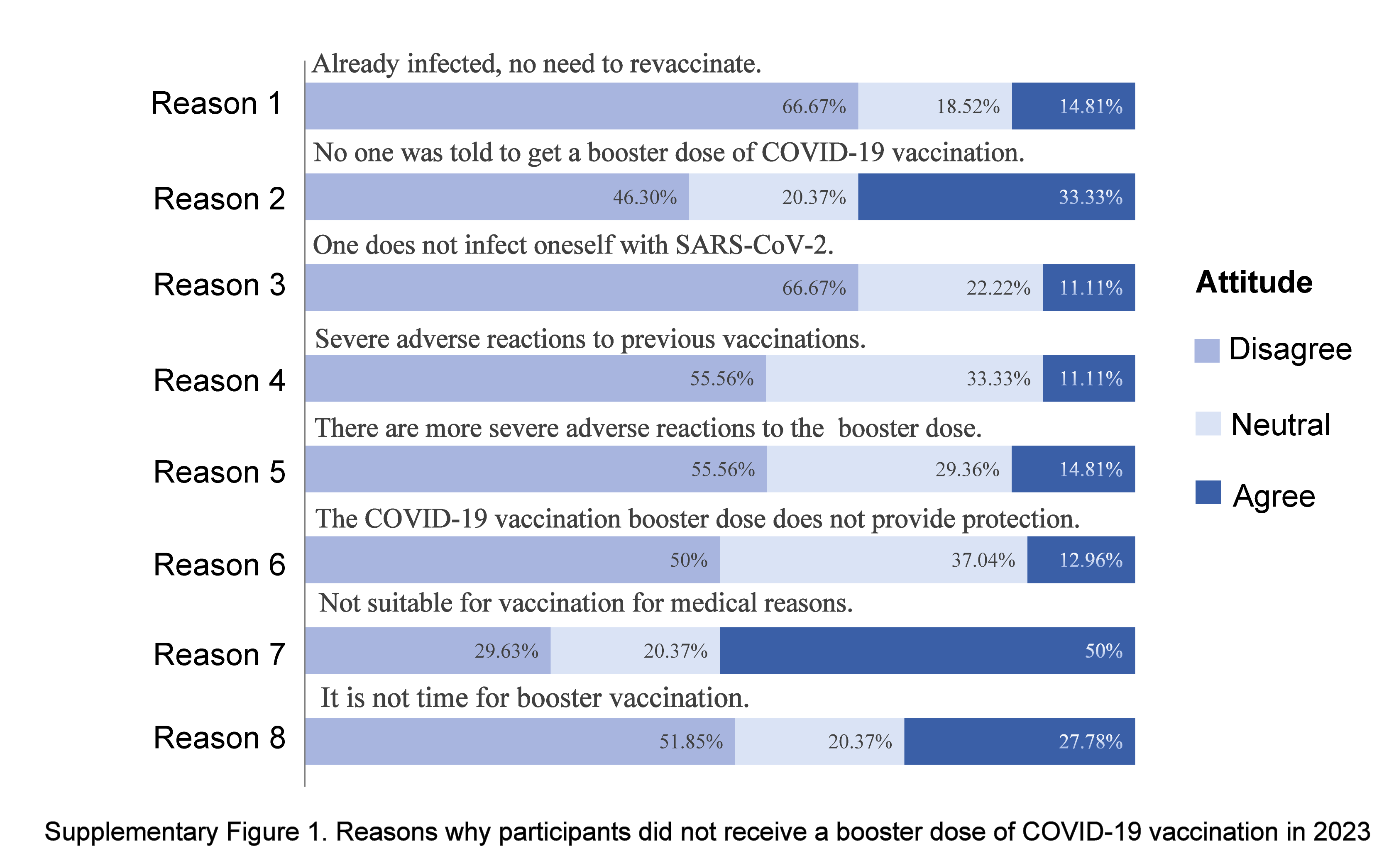

Supplement: Supplementary file 3 [file Image_1.tif]
